# Editorial: Homeostatic regulation of protein synthesis, folding and secretion by stress response pathways in eukaryotes

**DOI:** 10.3389/fcell.2023.1282272

**Published:** 2023-09-13

**Authors:** Advait Subramanian, Paolo Remondelli

**Affiliations:** ^1^ Department of Microbiology and Immunology, University of California, San Francisco, San Francisco, CA, United States; ^2^ G W Hooper Foundation, University of California, San Francisco, San Francisco, CA, United States; ^3^ Department of Medicine, Surgery and Dentistry “Scuola Medica Salernitana”, University of Salerno, Salerno, Italy

**Keywords:** homeostasis, protein secretion, protein quality control, stress response pathway, membrane trafficking

## Introduction

Homeostasis, in the strictest sense, means to maintain a dynamic process in a state of equilibrium and optimal functioning (Bernard, 1957; Cannon, 1987). To determine if a cellular process is homeostatically controlled, experimental perturbations have been carefully designed to induce a specific change to the process in question and observe its behaviour. Responses that are mounted to such perturbations depend not only on the process being perturbed, but also on where this process occurs within the cell, and how this process interacts with other, parallel, cellular processes (Barabasi and Oltvai, 2004; Stelling et al., 2004; Del Vecchio et al., 2022). The identification of such responses often results in a mechanistic understanding of the process by reverse engineering of its function (Csete and Doyle, 2002).

In eukaryotic cells, the control of protein quantity and quality is a fundamental process that is important for cellular, tissue and organismal life. Proteins in eukaryotic cells need to reach their correct intracellular or extracellular locations and to be properly modified in order to perform their functions efficiently and safely. This is achieved by an ensemble of organelles that fold, post-translationally modify, and deliver most of the transmembrane and nearly all of the secreted proteins—about a third of the human proteome—from their site of synthesis to their final cellular destinations where they are programmed to function.

It is thus not surprising that the regulation of cellular protein homeostasis is complex (Farhan and Rabouille, 2011; Sun and Brodsky, 2019; Del Vecchio et al., 2022). This Research Topic aims to explore this complexity and constitutes three review articles and one original research article that discuss the emergent pathways involved in protein secretion and protein folding and their roles in physiology and pathology.

## Regulation of protein secretion in the early secretory pathway

Secretory proteins are synthesized in the endoplasmic reticulum (ER), an organelle characterized by a network of tubular and cisternal membranes where cargo proteins are translocated and then folded (Sun and Brodsky, 2019). Properly folded cargo proteins are then packaged into vesicular carriers that bud from specialized sub-domains of the ER called ER exit sites. These cargo-laden carriers dissociate from the ER and leave for the Golgi complex (Lord et al., 2013; Raote et al., 2023). There, they are transported through the Golgi cisternae, whereby they are modified, mainly by glycosylation. They then reach the trans-Golgi network, where they are again sorted into specialized dissociative carriers and delivered to further cellular compartments including the basolateral and apical domains of the plasma membrane, the endo-lysosomal system or secreted into the extracellular space (Di Martino et al., 2019). The ER and the Golgi complex are also associated with specialized protein degradative pathways that eliminate defective products (such as misfolded proteins or aberrantly accumulated proteins) either via the proteasome or via the lysosome (Sun and Brodsky, 2019).

Notably, the cellular secretory pathway comprises not only of components that directly interact with cargo proteins to facilitate their packaging, transport, and post-translational modifications (Lord et al., 2013; Di Martino et al., 2019; Raote et al., 2023), but also on a regulatory network that supervises these activities through signaling mechanisms (Pulvirenti et al., 2008; Farhan and Rabouille, 2011; Jin et al., 2012; Centonze et al., 2019; Subramanian et al., 2019; Del Vecchio et al., 2022). Indeed, cells adapt to the flux of cargo containing carriers transiting through the secretory pathway (Del Vecchio et al., 2022), as well as to certain conditions of ER stress that reduce the abundance of COPII coated carriers, hence giving rise to alterations in cargo secretion and structural defects to post-ER compartments (Renna et al., 2006; Amodio et al., 2009).

In this series, Roberts and Satpute-Krishnan review the role of the protein transmembrane emp24 domain 9 (TMED9) as a cargo receptor in the early secretory pathway. The authors discuss how TMED9 might be implicated in various processes such as autophagy, lysosomal sorting, viral replication, and cancer. Tapia et al. discuss the intricate connection between autophagy and protein secretion. Interestingly, their review explores the possibility that during certain stress conditions, the activation of autophagy may require an active Golgi-to-ER retrograde transport.

## Role of the unfolded protein response in immunity

The ER possesses a repertoire of chaperones and enzymes that play a vital role in ensuring the correct folding of newly synthesized proteins into their tertiary or quaternary structures. The ER folding machinery is constantly challenged, especially in specialized secretory cells such as immune cells that synthesize and secrete large quantities of proteins (i.e., antibodies, cytokines, etc.). Furthermore, the control of protein folding homeostasis is crucial to maintain the biogenesis and the function of secretory pathway organelles. It is now well understood that a complex network of signaling pathways termed the unfolded protein response feeds back into the folding capacity of the ER (UPR^ER^) to ensure adaptation of cells to fluctuations of the levels of unfolded proteins (or the folding load) (Walter and Ron, 2011). Three ER transmembrane sensors control the activation of the UPR^ER^—the RNase/kinase inositol-requiring protein 1α IRE-1α, the protein RNA-like ER kinase PERK and the activating transcription factor 6 (ATF6) (Walter and Ron, 2011). The UPR^ER^ activates an adaptive response aimed at avoiding a presumably toxic build-up of unfolded proteins, thus preventing their secretion through, and/or aggregation within, the secretory pathway. Of these sensors, insights into the regulatory role of ATF6α remains less well understood. Addressing this gap, Gutiérrez-Ballesteros et al. report on the importance of ATF6α in regulating specific cytokine gene expression in dendritic cell populations activated with inflammatory stimuli. This study stimulates discussions on how ATF6α alone, or in concert with other components of the UPR^ER^, regulates an inflammatory response.

## Regulation of protein homeostasis in mitochondria

In eukaryotic cells, membrane-bound organelles called mitochondria are another major site of protein synthesis and folding. Mitochondria contain their own genomes (mtDNA) that encode for dedicated translation machinery components such as transfer RNAs and ribosomal RNAs, as well as thirteen proteins involved in oxidative phosphorylation (OXPHOS). The rest of the ∼1,200 mitochondrial proteins are encoded in the nucleus, translated on cytosolic ribosomes, and imported into mitochondria (Shpilka and Haynes, 2018).

When processes occurring in the mitochondria such as OXPHOS, mtDNA synthesis, translation, folding, or protein import are compromised, mitochondrial dysfunction ensues. In response, cells activate the mitochondrial unfolded protein response (UPR^mt^) to promote the repair and recovery of the mitochondrial network (Shpilka and Haynes, 2018). In recent years, progress has been achieved in deepening our understanding of the UPR^mt^. Addressing this topic, Ye et al. have meticulously examined a range of journal articles spanning from 2004 to 2022. Their analyses encapsulates the current state of knowledge and underscores emerging avenues for further studies.

## Conclusion

The articles presented in this Research Topic cover a wide range of homeostatic processes occurring in the ER, the Golgi, and the mitochondria. We hope that this Research Topic begins to inspire researchers to investigate stress response biology in development and disease from a modular systems perspective.
